# What happens to frequent attenders when they attend psychiatric liaison services? a clinical and demographic profile

**DOI:** 10.1192/bjo.2021.675

**Published:** 2021-06-18

**Authors:** Frederick Grose, Tennyson Lee, Richelle Canlas, William Phung, Rikke Albert, Alana Ahmet, Jia Song

**Affiliations:** East London NHS Foundation Trust

## Abstract

**Aims:**

We aimed to describe the demographic and clinical profile, and management of frequent attenders to a psychiatric liaison service.

**Background:**

Frequent Attenders to emergency departments contribute significantly to the burden on health services and by definition are subjectively highly stressed. It is therefore important that mental health services develop effective responses to this group of patients. A systematic literature search indicated a paucity of information on this group of patients.

**Method:**

We conducted a case series of 49 frequently attending patients to the Psychiatric Liaison service in Tower Hamlets, East London NHS Foundation Trust.

We defined frequent attenders as seeing the Psychiatric Liaison Service 5 or more times in 2018. We excluded 4 patients aged <18 years or >65 years.

For each patient we collected data regarding their demographics; the details of each attendance to the Psychiatric Liaison Service; and their use of other psychiatric services.

We then conducted a multivariate analysis, including stratification of patients based on number of attendances to identify correlation between frequency of attendance and the other information.

**Result:**

Demographic: The 45 patients reviewed had a mean age of 37 and a mean of 7 attendances during the study period.

Clinical: 89% had a history of emotional trauma, 71% of substance misuse, and 49% of any personality disorder. Only 9% of the patients were under the care of the locality Personality Disorder Service.

73% of the patients were under the care of any other psychiatric service. There was no correlation between being under other services and the frequency of attendance.

Only 31% had contact with the locality Frequent Attenders Service during the study period, as this was established recently.

**Conclusion:**

Psychiatric Frequent Attenders have complex needs, which do not fit neatly into existing psychiatric diagnoses and services.

The high frequency of emotional trauma, substance misuse and personality disorder indicates a need for training of clinicians in these services to manage these patients, as well as planning for referral pathways for this group of patients who provide services with major challenges in appropriate pathways to care and follow-up

